# An Interesting Surgical Case

**Published:** 1875-04-15

**Authors:** H. W. Sawtelle

**Affiliations:** Marine Hospital Service


### AN INTERESTING SURGICAL CASE.

Reported by H. W. Sawtelle, M.D., of the Marine Hospital Service.

PRIVATE George T. Cottrell, Co. G, 1st U. S. Sharpshooters, aged 21 years, was wounded while in the act of firing his piece, at the battle of Chancellorsville, Va., May 2, 1863, by a crusidal ball entering about half an inch above left clavicle, and about one inch from its sternal extremity, and passing transversely between the trachea and œsophagus, lodged beneath the clavicle near the point where the subclavian emerges. He was conveyed to Washington, D. C., and admitted to St. Aloysius Hospital May 7th, 1863.

There was but little blood lost. A numb pain supervened, which lasted six months, referred particularly to the elbow joint and fingers, the fingers remaining semi-flexed three months. He was unable to speak aloud for two weeks, and nourishment could only be taken in liquid form through a tube for several weeks. The forearm was carried at right angles, and narcotics were exhibited to relieve pain. By the middle of July following, the wound had perfectly healed, and never re-opened. At this date the joint, which had become firmly fixed at right angles from inaction, was straightened while the patient was under the influence of chloroform, and frequent flexion and extension subsequently restored fully the use of the joint.

He was returned to duty, in the Veteran Reserve Corps, Oct. 31, 1863, from whence he was discharged from the service Sept. 14, 1864, and pensioned.

The hand and fingers continued to be very sensitive to cold and heat, and at times intensely painful.

One morning in the fall of 1865, he found that the power of supporting the head was lost to such an extent, that he was unable to rise from his bed, and clonic contractions, drawing the head to the right shoulder, lasted four days, but did not recur.

Late in December, 1869, the pain at the point of lodgement of the missile increased, and January 31st, 1870, Professor N. S. Lincoln, M.D., having placed the patient under the influence of chloroform, cut down and removed the ball, which was found thrust in between the subclavian and a branch of the brachial plexus, the missile resting on the artery just where it emerges from beneath the clavicle, with the nerve drawn tightly across the ball in front. Upon pushing aside this nerve in order to remove the ball, vigorous contraction of the limb ensued. The wound healed readily.

The limb is now equal to its fellow in size and strength, and with the exception of a very slight sensitiveness of the fingers to cold and heat, which is improving, the patient is entirely relieved of his suffering.

---

ANTI-PYRETIC TREATMENT OF ACUTE RHEUMATISM.—The Mount Sinai hospital has adopted the treatment of acute rheumatism by the use of cold externally applied. The method consists in the use of cold baths, combined with ice-bags, to the inflamed joints. Every patient does not bear well the cold baths, but the ice-bags always prove grateful and always remove the pain. The very curious point has been noticed, that, if blankets are placed over the patient, or in any way the patient be allowed to sweat, the cold loses its efficacy. It is found also that, if the ice-bags are removed from the inflamed joints, the pain sometimes re-appears, and, when it does, a return to the ice-bags again relieves the patient.

